# Use of 90% ethanol to decontaminate stethoscopes in resource limited settings

**DOI:** 10.1186/s13756-017-0224-x

**Published:** 2017-06-15

**Authors:** Bijendra Raj Raghubanshi, Supriya Sapkota, Arjab Adhikari, Aman Dutta, Utsuk Bhattarai, Rastriyata Bhandari

**Affiliations:** KIST Medical College and Teaching Hospital, Imadol, Lalitpur, Nepal

**Keywords:** 90% ethanol, Cleaning, Decontamination, Isopropyl alcohol, Stethoscopes

## Abstract

**Background:**

In developing countries like Nepal, 90% ethanol is cheap and is available in most hospitals. The unavailability of isopropyl alcohol (IPA) in these settings led us to compare the efficacy between 90% ethanol and isopropyl alcohol pads in reducing the bacterial contamination of diaphragm of stethoscope.

**Methods:**

A randomized blinded experimental study was carried out to determine the difference between cleaning stethoscopes with 90% ethanol and IPA. Cultures of diaphragm were taken before and after cleaning with one of the cleaning agent. Colony forming units (CFU) count and organism identification was done by a blinded investigator. CFU before and after cleaning were compared using Wilcoxon signed–rank test. Mann Whitney U test was used to compare the decrease in CFU count between the cleaning agents.

**Results:**

About 30% of the stethoscopes harbored potential pathogens. Significant reduction in CFU was observed with both IPA (Wilcoxon signed–rank test, *P* value <0.001) and 90% ethanol (Wilcoxon signed–rank test, *P* value <0.001). Comparing median decrease in CFU between cleaning with IPA and with 90% ethanol, no significant difference was found (Mann Whitney U test; U = 1357, *P* value >0.05).

**Conclusions:**

Both 90% ethanol and IPA are equally effective in decontaminating the diaphragm of stethoscope. Selection of agent should be done on the basis of cost and availability.

## Background

Health care associated infections (HCAI) are more frequently seen in developing countries [1]. A meta-analysis has reported a prevalence of HCAI of 15.5 per 100 patients in developing countries, which is much higher than in developed countries [2]. Cross-transmission has been found to play an important role in nosocomial infection [3].

Stethoscopes are one of the most frequently used medical devices. Apart from normal skin flora, most stethoscopes harbor potential pathogens like *Staphylococcus aureus* (methicillin-sensitive or -resistant), *Klebsiella*, *Acinetobacter, Enterococcus* and extended-spectrum β-lactamase-producing *Enterobacteriaceae* [4–8]. Contamination of stethoscopes with methicillin-resistant *Staphylococcus aureus* (MRSA) ranges from 0% - 32% [9]. These potentially pathogenic organisms can survive on inanimate surfaces for days to months [10]. When stethoscopes are not decontaminated prior to use, they may act as vector [4–6, 8] leading to transmission of organisms from one patient to another.

Even after a single clinical examination, the level of contamination of stethoscope is not negligible [7, 11]. Diaphragms of stethoscopes get much more contaminated than the tubes [11]. Center for Disease Control recommends cleaning of stethoscopes when visibly soiled and after each use [12]. Both ethanol based cleansers and isopropyl alcohol significantly reduce the bacterial growth in culture [13].

Nepal is a developing country. Ethanol based cleansers or isopropyl alcohol pads are not usually available in resource limited hospitals of Nepal. However, 90% concentrated ethanol is easily available in these settings. Furthermore, it is cheaper than isopropyl alcohol and ethanol based cleansers. The objective of this study is to determine the effectiveness of 90% ethanol compared to isopropyl alcohol pads in reducing the bacterial contamination of diaphragm of stethoscope.

## Methods

### Study design

A randomized blinded experimental study was carried out from December 2016 to March 2017 at KIST Medical College and Teaching Hospital, a 700-bed tertiary care hospital in Nepal. The study was approved by Institutional Review Committee of KIST Medical College and Teaching Hospital.

### Participants

Participants were interns, medical officers, consultants and nurses from Intensive Care Unit, Emergency Room, Medicine, Surgery, Pediatrics and Gynecology/Obstetrics ward. A total of 108 stethoscopes were cultured before and after cleaning with either 90% ethanol or IPA. From each ward 18 samples were taken and half were cleaned with 90% ethanol and the other half with IPA. Randomization was done using the envelope technique. Stethoscopes used in this study belonged to the respective participants.

### Pilot study

A pilot study involving 6 participants was done before the study to determine an effective way to communicate and take samples. We found that participants were reluctant to clean the diaphragm imprinted on blood agar with bare hands. So a sterile gauze piece was introduced for cleaning with 90% ethanol.

### Intervention methods

The investigators went to the wards selected for study. After an informed written consent, participants were asked to draw a sealed envelope from the box. The size, color, texture and consistency of all the envelopes were similar in order to maintain concealment of allocation of cleaning agent. Inside of the sealed envelope was a piece of paper indicating the cleaning agent. Based on the envelope the participants had drawn, they were classified into 90% ethanol group (EG) and Isopropyl alcohol group (IPAG). The investigators provided instructions on aseptic technique of obtaining cultures. Then the participants plated the diaphragm of the stethoscope into the blood agar for 5 s with a firm but gentle pressure before and after decontamination. This culture method was used because it has been shown to be a quick, simple and efficient method [8]. IPAG participants used the IPA pad (70% Isopropyl Alcohol, Shanghai Yinjing Medical Supplies Co. Ltd) to wipe the diaphragm from center to periphery for a period of 5 s and then air-dried for 30 s before culturing again. EG participants were provided with a sterile gauze piece soaked with 4 ml of 90% ethanol obtained from a manual dispenser. They wiped the diaphragm from center to periphery for 5 s. The diaphragm was then air-dried for 30 s before culturing again. During the entire process, the investigators were present and provided step by step instructions to the participants. The stethoscopes were allowed to dry for 30 s because studies have shown that within 30 s both ethanol [14] and isopropyl alcohol [15] kill 99.9% of the bacteria on skin and hard surfaces.

### Laboratory analysis

Stethoscopes were cultured on laboratory made blood agar. Blood agar was prepared in the laboratory with 5% *w*/*v* sterile defibrinated blood and agar base (HiMedia Laboratories Pvt. Ltd) according to manufacturer’s guidelines. After sterilization, the medium was cooled to 50 °C, supplemented with 5% *w*/*v* sterile defibrinated blood, mixed well and poured into sterile petri plates. After preparation of blood agar, samples from each batch were tested for quality control and stored at 2 – 8 °C.

Stethoscope cultures were incubated at 37 °C for 48 h in biochemical oxygen demand incubator under aerobic conditions and then observed for growth. Colony Forming Units (CFU) count was done by a blinded investigator. The organisms were identified by manual method using colony morphology, staining reaction (Gram stain) and standard biochemical tests (catalase test, coagulase test, oxidase test, triple sugar iron agar test, citrate utilization test, urease production test and sulfur indole motility test). The genus of gram positive rods were not further identified as these organisms are considered skin contaminants with low pathogenic potential. *Staphylococcus aureus* isolates were further cultured on Mueller Hinton agar for disk diffusion testing using Clinical and Laboratory Standards Institute (CLSI) guidelines (M2-A9) [16]. Resistance to cefoxitin (30 μg) was used to detect MRSA using CLSI guidelines (M 100-S16) [17].

### Statistical analysis

As our results did not follow a normal distribution, we used nonparametric tests for statistical analyses. Median decrease in CFU after cleaning with IPA and 90% ethanol were compared using the Mann Whitney U test. For Mann Whitney U test, power analysis using GPower 3.1.9.2 indicated that at least 47 samples were required in each group to differentiate an effect size of 0.5 at 95% CI with power > 0.8. For each cleaning agent, Wilcoxon signed-rank test was used to determine the difference between baseline CFU and post cleaning CFU. Statistical analyses were done using IBM SPSS Statistics 23.

## Results

**Fig. 1:**
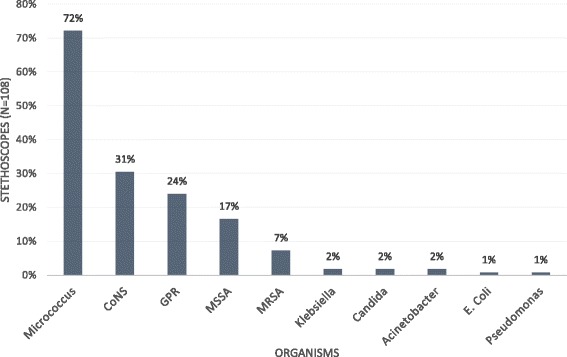
Frequency of isolation of organisms from stethoscopes (*n* = 108). MRSA, Methicillin-resistant *Staphylococcus aureus*; MSSA, Methicillin-sensitive *Staphylococcus aureus*; GPR*,* Gram Positive Rods; CoNS, Coagulase negative *Staphylococcus*

Out of 108 stethoscopes cultured, 105 (97.2%) cultures yielded at least 1 CFU. Figure 1 shows the various organisms isolated. Commonly isolated organisms were *Micrococcus*, Coagulase negative *Staphylococcus* (CoNS), gram positive rods (GPR) and *Staphylococcus aureus*. *Micrococcus*, CoNS and GPR were considered as skin contaminants with low pathogenic potential. Other microorganisms isolated like *Staphylococcus aureus*, *Klebsiella*, *Acinetobacter*, *Candida, E. coli* and pseudomonas were considered as potential pathogens. Potential pathogenic organisms were isolated from 33 stethoscopes (30.5%). MRSA (methicillin-resistant *Staphyloccus aureus*) was present in at least one stethoscope from every ward studied. Potential pathogenic organisms obtained from Intensive care unit (ICU) were *Klebsiella*, *Acinetobacter*, *Candida* and *Staphylococcus aureus*.

**Fig. 2:**
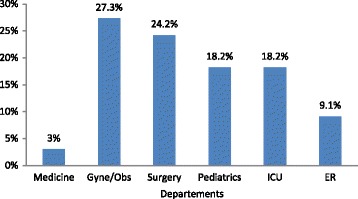
Proportion of stethoscopes contaminated with potential pathogenic organisms before decontamination in each ward. Total number of stethoscopes studied in each ward was 18

Figure 2 shows the proportion of stethoscopes contaminated with potential pathogenic organisms before decontamination in each ward. Higher proportion of stethoscopes in surgery (24.2%) and gynecology/obstetrics (27.3%) ward were contaminated with potential pathogenic organisms. There was significant difference in proportion of stethoscopes contaminated with potential pathogenic microorganisms across the wards (X^2^ = 11.913, df = 5, *P* = 0.036).

After cleaning with IPA or 90% ethanol, 96 (88.88%) stethoscopes were growth free. Even though cleaning with 90% ethanol resulted in higher percentage (92.6%) of growth free stethoscopes compared to IPA (85.2%), the difference was not significant (X^2^ = 1.50, *p* = 0.221). Only 12 (11.1%) stethoscopes resulted in growth after decontamination. *Micrococcus*, CoNS and GPR were isolated from 7, 2, and 3 stethoscopes respectively. Methicillin-sensitive *Staphyloccus aureus* (MSSA) was isolated from 1 stethoscope.

Table 1 compares the baseline median CFU and post cleaning median CFU. Significant reduction in CFU was observed with both IPA (Wilcoxon signed-rank test, *P* value <0.001) and 90% ethanol (Wilcoxon signed –rank test, *P* value <0.001). Comparing median decrease in CFU between cleaning with IPA and with 90% ethanol, no significant difference was found (Mann Whitney U test; U = 1357, *P* value = 0.535) (Table 2).

**Table 1:** Comparison between baseline median CFU and post cleaning median CFU for each cleaning agent

Cleaning agent	Baseline Median CFU (IQR)	Range of Variation	Post cleaning Median CFU (IQR)	Range of variation	Percent reduction in CFU (mean)	*P* value for Wilcoxon signed-rank test
IPA	22.5 (7–48)	0–110	0 (0–0)	0–21	93.14%	<0.001
90% ethanol	17.5 (7–31)	0–106	0 (0–0)	0–5	97.60%	<0.001

**Table 2:** Comparison of median decrease in CFU between IPA and 90% ethanol

Cleaning agent	Median decrease in CFU (IQR)	Range of variation	Mann Whitney U test (U)	*P* value
IPA	20.5 (7–48)	0–108	1357	0.535
90% ethanol	17.5 (7–31)	0–105		

## Discussion

One third of the stethoscopes resulted in growth of potential pathogenic organisms. This supports previous findings in literature that stethoscopes harbor potential pathogenic organisms [4–8]. *S. aureus* is the most common potential pathogenic organism isolated from stethoscopes [5, 6, 8, 18]. Similarly in our study, MSSA and MRSA were the most common potential pathogenic organisms isolated (Fig. 1). MRSA was isolated from 7% of the stethoscopes. Use of such stethoscopes without decontamination may result in colonization of MRSA in a new patient. Organisms like *Acinetobacter* and *Klebsiella* have been isolated from stethoscopes of ICU. These organisms are often multidrug resistant and difficult to treat. Stethoscopes acting as vector may add to worry of critically ill patients in ICU. The CFU obtained in our study (median [IQR], 22.5 [7–48] and 17.5 [7–31]) is comparable to findings from other studies (median [IQR], 10 [3–42]) [18], (median [range], 22 [3–300] and 15 [1–200]) [8].

Higher proportion of stethoscopes in surgery (24.2%) and gynecology/obstetrics (27.3%) ward were contaminated with potential pathogenic organisms. These wards treat patients with wounds, burns, catheters and other invasive procedures. Cross-transmission with stethoscopes in these wards may pose a risk for nosocomial infection. Stethoscopes from medicine ward were relatively free from potential pathogenic organisms. Only 1 stethoscope was found to contain such organism. Significant difference was detected in proportion of stethoscopes harboring potential pathogenic organisms in different wards. Previous study from tertiary care hospital of Nepal reported higher bacterial contamination of stethoscopes in surgery, ER and gynecology wards [19]. The same study identified highest bacterial contamination within 15 min of last use. Time since last use of stethoscope was not considered in our study which may have accounted for our finding. There is a significant direct correlation between isolation of potential pathogens from stethoscope and physician’s hand [7]. Although our hospital has a hand hygiene policy, the data on adherence to hand hygiene is not available. Differences in compliance to hand hygiene across the wards may have accounted for our finding. We did not consider time since last decontamination which may also have influenced our finding as decontamination significantly reduces bacterial growth in culture [13]. In contrast to our finding, Shiferaw T et al. found no significant difference in isolation of potential pathogens from different wards [20].

Health care professionals often neglect cleaning of stethoscopes. Previous surveys reveal that 7% – 45% of the healthcare professionals from different wards had never cleaned their stethoscopes [4, 21]. After clinical examinations, the contamination of stethoscope is comparable to physician’s hand [11]. So cleaning of hands should be accompanied by cleaning of stethoscopes, before and after each patient contact. Our institution lacks policy on decontamination of stethoscopes between individual patients. We recommend inclusion of stethoscope decontamination in the infection control policy.

Pilot study revealed that participants were reluctant to touch the diaphragm imprinted on blood agar with bare hands. So the study was designed to provide a sterile gauze piece soaked with 90% ethanol for EG participants. However, in clinical settings where stethoscopes are not visibly soiled, we recommend direct placement of 90% ethanol in diaphragm and rubbing its surface with hands. This would limit the use of valuable resource and reduce generation of waste.

Both IPA and 90% ethanol significantly reduced CFU as well as successfully eliminated potential pathogenic organisms. Only 1 stethoscope resulted in growth of potential pathogenic organism (MSSA) after decontamination with these agents. No statistical difference was found between cleaning with 90% ethanol and IPA. These results are consistent with similar study comparing IPA with ethanol-based cleanser [13]. Selection of agent should be based on the availability and cost. In developing countries like Nepal, 90% ethanol would be a good agent as it is readily available and cheaper than IPA. For those using IPA, wiping the diaphragm surface from center to periphery for 5 s should successfully decontaminate the surface. We believe that obtaining 90% ethanol or ethanol based cleansers from a dispenser is less time consuming than obtaining IPA pad from its sachet. Decontamination with ethanol based cleansers are also suggested to be more convenient as it requires no extra time than standard hand sanitization [13].

There were several limitations of our study. Apart from *S. aureus*, identification of other organisms to species level and their antimicrobial susceptibility testing was not carried out as this would require more resources and was not a part of our study protocol. Our findings were based on a single hospital. However, we believe our hospital is similar to other teaching hospitals and government tertiary care hospitals of Nepal in terms of resources and infection control policy. Thus our findings can be generalized to these hospitals. Generalizability of our findings to private care settings may be limited due to their extensive resources and dedicated infection control policy. Time since last use and cleaning of stethoscopes were not considered while taking samples which could have accounted for difference in isolation of potential pathogenic organisms from different wards. As CFU count was done by direct visual assessment, there may have been small error in counting CFU when high numbers of CFU were obtained. However, this error would have minimal effect when comparing IPA and 90% ethanol because the error would be evenly distributed between IPAG and EG and would uniformly affect decrease in CFU for both agents. In future studies, a more precise CFU count can be obtained by using automated method [22] or taking a digital photograph of culture plate and using image editing software to count the CFU [11].

## Conclusions

Stethoscopes harbor potentially pathogenic microorganisms. Both IPA and 90% ethanol are effective in decontaminating stethoscope. There is no difference in cleaning of stethoscope diaphragm with 90% ethanol or IPA. Selection of agent should be done on the basis of cost and availability.

## Abbreviations

CFU: Colony forming units
CLSI: Clinical and laboratory standard institute
CoNS: Coagulase negative *Staphylococcus*
EG: Ethanol group
ER: Emergency room
GPR: Gram positive rods
Gyne/Obs: Gynecology and obstetrics
HCAI: Health care associated infections
ICU: Intensive care unit
IPA: Isopropyl alcohol
IPAG: Isopropyl alcohol group
IQR: Inter quartile range
MRSA: Methicillin-resistant *Staphylococcus aureus*
MSSA: Methicillin-sensitive *Staphylococcus aureus*
